# Evaluation of the Occurrence of False Aneurysms During Halal Slaughtering and Consequences on the Animal’s State of Consciousness

**DOI:** 10.3390/ani10071183

**Published:** 2020-07-13

**Authors:** Giancarlo Bozzo, Elisabetta Bonerba, Roberta Barrasso, Rocco Roma, Francesco Luposella, Nicola Zizzo, Giuseppina Tantillo

**Affiliations:** 1 Department of Veterinary Medicine, University of Bari, Strada Provinciale per Casamassima km 3, 70010 Valenzano (BA), Italy; giancarlo.bozzo@uniba.it (G.B.); elisabetta.bonerba@uniba.it (E.B.); nicola.zizzo@uniba.it (N.Z.); 2 Department of Agricultural and Environmental Science, University of Bari, Via Orabona 4, 70126 Bari, Italy; rocco.roma@uniba.it; 3 Postgraduate School in Inspection of Food of Animal Origin, Veterinary Medicine, University of Bari, Strada Provinciale per Casamassima km 3, 70010 Valenzano (BA), Italy; francescoluposella@hotmail.it; 4 Interdisciplinary Department of Medicine, University of Bari, Piazza Giulio Cesare 11, 70124 Bari, Italy; giuseppina.tantillo@uniba.it

**Keywords:** animal welfare, consciousness, false aneurysm, Halal slaughtering

## Abstract

**Simple Summary:**

False aneurysm of the severed arteries is a potential concern in animals as it could extend the duration of brain function before they die. Indeed, false aneurysm formation in the carotids and a slow rate of blood loss may extend the period of consciousness during slaughter without stunning. The aim of this study was to monitor the development of false aneurysms in animals slaughtered using Halal procedures and to evaluate the possible link between the onset of false aneurysm, the extension of the bleeding phase, and the persistence of signs of consciousness in the animal. We analysed, also using a histological test, the correlation between false aneurysms and the location of the cut made by the operator at the neck, which varied between the first and the fourth cervical vertebrae. Performing the cut at the first cervical vertebra reduced false-aneurysm formation and decreased the period of consciousness of Halal-slaughtered animals without pre-stunning. This could prevent unnecessary suffering, welfare abuse, and the failure of properly bleeding.

**Abstract:**

This study evaluated the occurrence of false aneurysms and reflexes in bovines, associated with the point along the neck where the cut was performed. The survey was carried out on a total of 1200 male beef cattle, belonging mainly to the Charolais and Limousin breeds, aged between twelve and twenty-four months. In the slaughterhouse, three operators, identified by convention as Operator C4, Operator C2, and Operator C1, performed the Halal slaughtering. Each operator carried out the slaughter of 400 animals and, on the basis of his own professional training, performed the cutting of the vessels at different points along the neck: Operator C4 between the third and fourth cervical vertebrae; Operator C2 at the level of the second cervical vertebra; and Operator C1 at the first cervical vertebra. The occurrence of false aneurysms was assessed on the basis of the different cutting site used by the three operators. Then, the evaluation of consciousness indicators, that is, rhythmic breathing and eye response, closely related to a slow bleeding process and to a delayed loss of brain function, was carried out 90 s post-bleeding. The group of cattle slaughtered by Operator C4 had a prevalence of false aneurysms of 10.25%. Conversely, the other two groups of cattle slaughtered by Operators C2 and C1 showed false aneurysm formation in 7.25% of each case. Further, 37.5% of the animals (18 for Operator C4, 17 for Operator C2, and 16 for Operator C1) with signs of consciousness 90 s after sticking were consequent to the onset of false aneurysms and, more precisely, they were owing to a late second cut of the vessel carried out by the operator, when false aneurysms occurred. The results of the research showed that the cutting point chosen by the operator is a key element in determining the onset of false aneurysms.

## 1. Introduction

Ritual slaughters in the European Union are performed by way of a derogation from the requirements of Council Regulation (EC) 1099/2009 [1] on the protection of animals at the time of killing. Slaughter without stunning is recognised as a religious prerogative, but there are concerns that the methods can compromise animal welfare. One of these concerns regards the loss of consciousness because, without pre-stunning, the animal could experience more pain and/or distress following the cut [2].

Correct cutting of the neck and vessels (or only vessels) serves a dual function: (i) causing the animal to die as quickly as possible (by inducing acute anaemia) and (ii) preserving the qualities of future meat products [3]. Indeed, bleeding causes a loss of consciousness linked to brain anoxia and reduction of glucose intake, but these effects are frequently less rapid in animals slaughtered according to a religious rite [4,5]. On the other hand, a correct exsanguination of the animal is essential because it improves quality and extends the shelf life of the meat intended for human consumption [6]. Moreover, a greater amount of retained blood leads to poor meat quality [7,8] and blood is a perfect medium for the growth of bacteria [9].

False aneurysm formation in the cardiac ends of the severed arteries prolongs consciousness in cattle slaughtered without stunning [10,11]. In fact, cattle have a particular anatomical conformation of the blood vessels [12,13] that allows for collateral blood supply to the brain through a basi-occipital plexus, in addition to branches from the carotid and basilar arteries. This plexus allows blood to pass to the brain through an alternative route to the branches of the carotid arteries [14] and delays cerebral hypoxaemia during occlusion of the carotids [15,16].

False aneurysm of the severed arteries is a potential concern in animals, as it could extend the duration of brain function before the animals die [17]. In ballooned arteries, there is coagulated blood between the outer surface of the artery and the inner aspect of the connective tissue sheath surrounding the artery, suggesting the formation of a false aneurysm [17]. The resulting encapsulated haematoma can range from a uniform concentric layer around the artery to an irregular bulge, which creates tortuosity in the artery [13]. It is clear that false aneurysm formation in the carotids, collateral blood flow through the plexuses, and a slow rate of blood loss may extend the period of consciousness during slaughter without stunning [18,19]. In order to ensure that the animal is unconscious, the absence of ocular reflexes needs to be associated with other indicators of unconsciousness, for example, rhythmic breathing; indeed, the presence of breathing after stunning is an indicator of the need to proceed to a second stunning [20,21].

A study [22] carried out on calves of different ages has shown that the duration of loss of consciousness or probable death (isoelectric electroencephalogram varies between 5 and 336 s. In adult cattle [18], the duration of unconsciousness varies between 7.5 and 77 s [23,24]. In a study conducted by Gregory et al. [6], seventy-one percent of the cattle that took more than 75 s to collapse had false aneurysms in the cardiac ends of the severed carotid arteries. Moreover, false aneurysm in the cardiac and cephalic ends of the severed carotid arteries was the major reason for prolonged (≥60 s) consciousness of slaughtered animals. False aneurysm can form as early as 7 s and on average within 21 s following Halal slaughter, leading to sustained consciousness due to failure to bleed out properly [25].

Therefore, the aim of this study was to monitor the development of false aneurysms in animals slaughtered using Halal procedures. We also tried to evaluate the possible link between the onset of the false aneurysm, the extension of the bleeding phase, and the persistence of signs of consciousness (rhythmic breathing and eye response). We analysed the correlation between false aneurysms and the position at which the cut was performed by the operator, which varied between the first and fourth cervical vertebrae. We also carried out a histological test to highlight any possible differences in the carotid arteries caused by the point of the cut. Finally, we evaluated the operator’s reactivity in cutting the vessel and their behaviour in resolving such events.

## 2. Materials and Methods

### 2.1. Sampling

The study, resulting from a partnership between the Food Safety Section of the Department of Veterinary Medicine at Bari University (Italy) and a slaughterhouse practicing Halal slaughtering, in Apulia region (Southern Italy), was conducted in the period between August 2017 and August 2019. Data were provided by the slaughterhouse, without our involvement in their collection. The survey was carried out on a total of 1200 male beef cattle, belonging mainly to the Charolais and Limousin breeds, aged between twelve and twenty-four months.

After transport of about one hour, all the animals arrived at the slaughterhouse, where they were kept in the lairage facilities until slaughter. The authorized slaughtermen restrained the animals in a full-inversion rotary pen for presenting the animal in dorsal recumbency for the cut. The head was completely restrained to avoid any movement of the animal that could compromise the effectiveness of the cut. After this step, the animals were slaughtered according to Islamic religious ritual (without stunning) using a knife through the structures at the front of the neck: trachea, oesophagus, carotid arteries, and jugular veins, using various cutting techniques.

In the slaughterhouse, three operators, identified by convention as Operator C4, Operator C2, and Operator C1, performed the Halal slaughtering. The three operators had attended the same training course and had the same number of years of service in that slaughterhouse. Each operator carried out the slaughter of 400 animals and, on the basis of his own professional training, cut the vessels at different points along the neck: (i) Operator C4 made the cut between the third and fourth cervical vertebrae (caudal cut C3–C4); Operator C2 made the cut at the level of the second cervical vertebra (intermediate cut C2); and Operator C1 made the cut at the first cervical vertebra (cranial cut C1). For each of the three operators, we divided the 400 animals into 10 observations, each consisting of 40 slaughters.

The onset of false aneurysms (FA) was evaluated by the operators, firstly by observing the macroscopic vessels in which the formation of a clot occurred, leading to slow bleeding and, secondly, by assessing whether the period of consciousness of the animals increased. The assessment of the occurrence of FA was carried out on the basis of the different cutting site performed by the three operators. False aneurysms were considered resolved if, following the second cut made by the operator, an appropriate bleeding of the animal was observed, without the onset of reflexes.

Evaluation of the animal’s consciousness indicators, that is, rhythmic breathing (RB) and eye response (ER), closely related to a slow bleeding process and to a delayed loss of brain function, was carried out by the three operators 90 s post-bleeding. The choice to wait 90 s before assessing reflexes was established in the health control plan of the slaughterhouse. Indeed, according to the Italian standard operating procedures for monitoring animal welfare at the slaughterhouse [26], the absence of the corneal reflex should be evaluated between 1 and 2 min after cutting the neck. The animals were restrained in the full-inversion rotary pen for all 90 s, after which they were hoisted and the total loss of consciousness of the animals was verified by the operators. The corneal reflex was tested by lightly touching the cornea and, if present, the eyeball would retract slightly, and the eyelids would close. The eyelid reflex was tested by slightly touching the eyelid. Whereas it was difficult to separate positive corneal and eyelid reflexes during slaughtering, as their neuronal circuit is largely similar, a generalised eye response was recorded, which was a combination of the corneal and eyelid reflexes. Both RB and ER were evaluated by the religious responsible for the Halal Committee, without any type of instrumental evaluation.

### 2.2. Histological Test

At the slaughterhouse, carotid artery samples were collected from 15 bovines (5 per operator) in which false aneurysms were found. These samples were fixed in 10% neutral buffered formalin for the evaluation of possible vascular alterations. At the pathological anatomy unit of the Department of Veterinary Medicine at the University of Bari, the samples were embedded in paraffin wax, sectioned at 4 µm, and processed for histological examination. The sections were stained with (i) Haematoxylin–Eosin (H.E.) to characterize anatomical structures; and (ii) Mallory's trichrome, in order to highlight the elastic fibres. Each section was thus assessed for wall thickness and lumen area, as well as structural relations between connective tissue, blood clot, and artery wall.

### 2.3. Statistical Analysis

The prevalence of false aneurysms was obtained using descriptive statistics of the data. The observations were elaborated in order to evaluate which of the three operators showed higher numbers of false aneurysm formations in the slaughtered animals and whether these false aneurysms were followed or otherwise by the presence of RB, ER, or both. Moreover, in relation to the occurrence of the reflexes, the percentage of animals rejected per operator was calculated. The mean values of the three groups (Operators C4, C2, and C1) and of the three different situations (total number of FA, resolved FA, and unsolved FA) valuated in this study were compared by one-way analysis of variance (ANOVA). Data were analyzed using IBM SPSS (Statistical Package for the Social Science, NY, USA) version 21. *p*-values < 0.05 were considered statistically significant.

## 3. Results

The total prevalence of FA among the three operators was of 8.25% (99 cases out of 1200 animals). The group of cattle slaughtered by Operator C4 had an FA prevalence of 10.25%. Conversely, the other two groups of cattle slaughtered by Operators C2 and C1 showed FA formation in 7.25% of cases. Furthermore, Operator C4 correctly resolved 23 FA out of 41 (56.10%); Operator C2 12 out of 29 (41.38%); and, finally, Operator C1 13 out of 29 (44.83%) (Table 1).

ANOVA results showed that in the case of both the total number of FA and of resolved FA, the difference between Operator C4 and Operator C2 was significant (F: 98.12; *p* < 0.001 and F: 75.21; *p* < 0.001, respectively), as was that between Operator C4 and Operator C1 (F: 89.50; *p* < 0.001 and F: 62.15; *p* < 0.001, respectively). On the other hand, the difference between Operator C2 and Operator C1 was not significant in either the total number of FA (F: 0.185; *p* < 0.001) or in the resolved FA (F: 0.622; *p <* 0.001) (Table 2 and Table 3). Regarding the unsolved FA, there was no statistical difference among the three Operators C4, C2, and C1 (Table 4).

**Figure 1:**
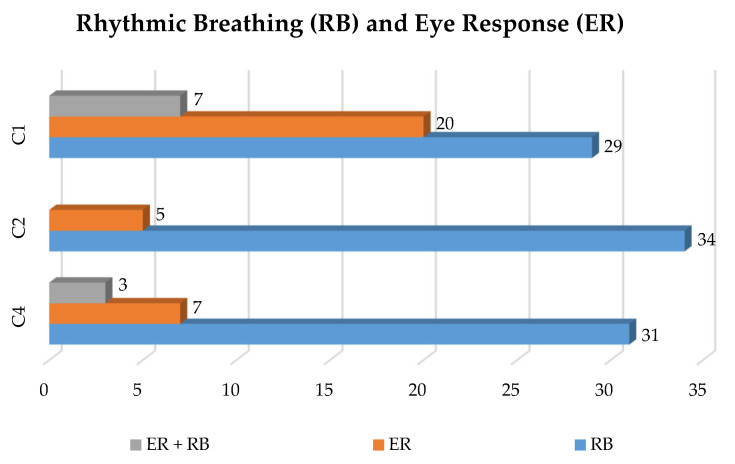
Number of animals with rhythmic breathing (RB) and eye response (ER) or both (RB + ER) in the 400 animals assessed by each operator (C4, C2, and C1).

Regarding the presence of reflexes, whose distribution is represented in Figure 1, Table 5 shows that most of the reflexes were independent of FA formation. Only a few reflexes were consequent to the onset of FA, and such cases were owing to a late second cutting of the vessel by the operator.

The percentage of rejected animals out of the initial number of 400 was similar for Operator C4 (10.25%) and Operator C2 (9.75%), while it was higher for Operator C1 (14%). Conversely, the error rate for the three different operators in resolving the FA promptly, before the onset of reflexes, was almost identical for the initial 400 animals (4.5% for Operator C4, 4.25% for Operator C2, and 4% for Operator C1).

### Histopathology

**Figure 2:**
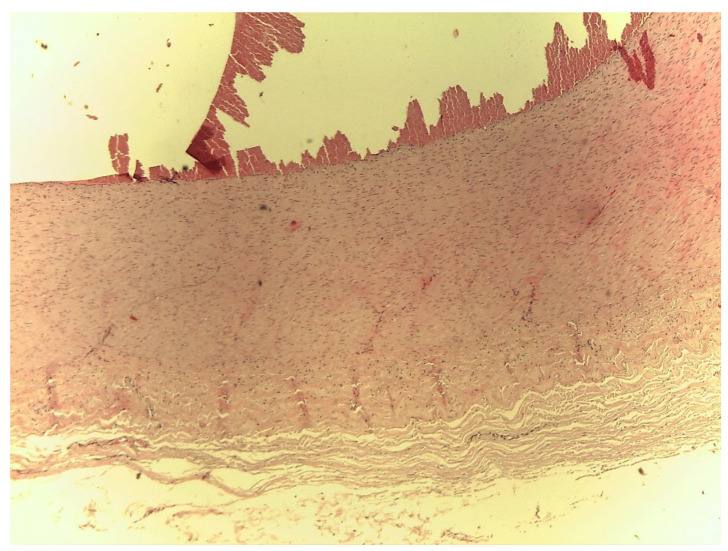
Bovine carotid artery with false aneurysm. Haematoxylin–Eosin (H.E.) (20×).

**Figure 3:**
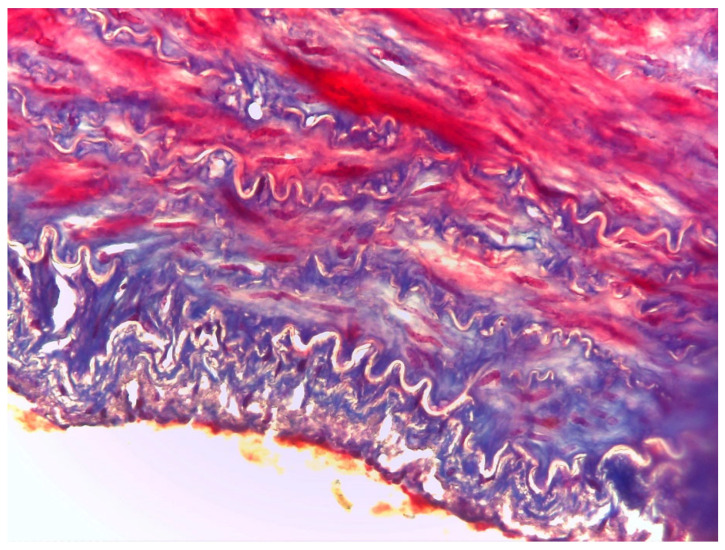
Bovine carotid artery with false aneurysm. Mallory's trichrome (40×).

The histological test showed no differences between the three different neck cutting locations. In general, fragments of a clot could be seen lying on the vascular endothelium, arranged in an irregular fashion (Figure 2) and evident distension of the elastic and muscular fibres, characterized in some areas by irregularity and interruption (Figure 3). Moreover, swelling at the distal end of the artery was associated with erythrocytes and fibrin trapped within the connective tissue sheath surrounding the artery. On the other hand, there were no signs of blood infiltration within the artery wall, suggesting that this formation was not a true aneurysm.

## 4. Discussion

The results need to be interpreted with caution because our study was limited by several factors. First of all, we used information provided by the slaughterhouse and we were not involved in the data collection. The slaughterhouse reports did not specify some aspects such as the exact time of false aneurysms or the onset of reflexes, as well as the exact number of the slaughtered animals belonging to the two breeds and the days when the slaughters took place. Consequently, our data cannot be used to compare the activity of the three operators in terms of quality, but only of quantity. Secondly, the onset of false aneurysms may have been influenced by the operators because, even though the three operators had attended the same training course and worked for the same amount of time in that slaughterhouse, their cut may be affected by each one’s manual skills. In a future study, it would be more appropriate to consider a single operator who makes cuts at different points along the neck in order to totally eliminate the variability linked to the operator. Another limit of our study is its failure to compare Halal and traditional slaughtering in order to verify the importance of stunning in preventing the onset of false aneurysms and reflexes and, consequently, in safeguarding and preserving animal welfare. Comparison with bovines slaughtered with the traditional method was impossible because these animals, after being stunned by captive bolt gun, were jugulated with a cut at the entrance of the chest, so no false aneurysms occurred. Notwithstanding these limitations, this manuscript reports the occurrence of false aneurysms and reflexes in bovines associated with the point along the neck where the cut was performed, thus increasing our knowledge of animal stress during slaughter.

In this study, we evaluated the occurrence and the prevalence of false aneurysms during Halal slaughter, the promptness of the operator to perform a second cut of the neck, and the percentage of rejected carcasses. The results of the research showed that the point along the neck where the cut was made is a key factor in determining whether false aneurysms occur [25]. Indeed, the cattle slaughtered by Operator C4, who performed the most caudal cut (between C3 and C4), presented a higher onset of false aneurysms (10.25%) than the animals jugulated by Operators C2 and C1, which showed exactly the same percentage onset of false aneurysms (7.25%), corresponding to 29 out of 400 animals slaughtered. The slight difference between operators C2 and C1 in the onset of FA and in resolving them is particularly evident when also considering the ANOVA results.

Our findings are in line with the results of Gregory et al. [25], who showed that cutting the neck at the level of the first cervical vertebra, instead of between the second and fourth vertebrae, reduced the likelihood both of false aneurysms and the early arrest of blood. Indeed, the risk of clot formation is lower when the knife is oriented towards the first cervical vertebra, rather than towards the second or third [27], while cutting the neck at the level of the third cervical vertebra increases the probability of early stoppage of the blood flow by fourfold [27]. Furthermore, cutting the neck at the first cervical vertebra would reduce the risk of irritation associated with contamination of the respiratory tract with blood [25]. This situation is the result of the cutting of both laryngeal nerves, which transmit sensory signals from the upper part of the respiratory tract, and the vagus nerve, which transmits signals from the lungs and inferior trachea [28]. In accordance with Gregory et al. [27], the reduced formation of false aneurysms when the cut is performed at the level of the first cervical vertebra can be explained considering that the common carotid artery has more branches at the C1 level, hence the risk of false aneurysms is lower than between positions C2 and C4. Moreover, the reduced formation of false aneurysms is linked to a lower presence of connective tissue around the carotid arteries at C1 level than between positions C2 and C4 [17]. Our study also highlights a greater onset of false aneurysms as a consequence of a more caudal cut at the level of the neck, compared with the execution of a central or cranial cut. However, false aneurysms were occasionally reported when arteries were severed at the C1 position [27], but they were usually caudal to the severed end of the artery and did not interfere with blood flow from the main artery.

As reported by Blackmore [18] and later by Bager et al. [29], the presence of false aneurysms could increase the risk of the animal regaining consciousness. Indeed, the swelling of the carotid artery and the slow rate of exsanguination may prolong the period of consciousness during slaughter without stunning. These dangers are increased in cattle because they have a collateral blood flow to the brain through an occipital-vertebral anastomosis, which may allow an extended perfusion of the brain [14].

Our study highlights that Operator C4 solved a greater number of false aneurysms (56.10%) compared with Operators C2 (41.38%) and C1 (44.83%). This difference can be explained considering that Operator C4 was quicker and more careful in identifying the formation of false aneurysms immediately after cutting or he was better used to dealing with a similar situation. Indeed, ANOVA results showed that the total number of unsolved false aneurysms differed only very slightly between the three operators (18 for Operator C4, 17 for Operator C2, and 16 for Operator C1), starting from a very different number of false aneurysms (41 for Operator C4 and 29 for Operators C2 and C1). This could be linked to either greater experience of Operator C4 in resolving cases of false aneurysm formation, or it is possible that, by cutting between the third and the fourth cervical vertebrae, the swelling of the carotid artery can be observed more quickly.

Of the 400 animals slaughtered by each operator, there were 41 cases of onset of reflexes for Operator C4, 39 for Operator C2, and 56 for Operator C1. These reflexes can be divided into those consequential to and those independent of false aneurysms, which are related to individual differences in the time of onset of consciousness. Those consequential to false aneurysms are linked to operator error in making the second jugulation cut, which was either too late or performed incorrectly. Conversely, the reflexes independent of false aneurysms cannot be controlled by the operator because the onset of rhythmic breathing and/or eye response may also depend on the animal’s level of agitation at the time of the neck cut and on the breed of the animal itself. Indeed, there are animals with an excitable temperament and animals with a docile temperament, which could show different cortisol levels when restrained [30].

Given that we cannot act on reflexes linked to an animal’s emotive state, we should concentrate on trying to eliminate or minimize reflexes consequential to false aneurysms and, therefore, on the cut performed by the operator. For this purpose, as the point of the jugulation cut during Halal slaughter is not indicated in a precise and rigorous way, it is recommended to carry out the cut where there is less probability of consequences for the animal and, therefore, at the level of the first cervical vertebra.

This is in accordance with a *hadith* that requires the cut to be made on the neck, just below the gullet and the core of the neck. The *hadith* further states that the jugular veins and carotid arteries must be cut, in addition to the oesophagus and trachea [31]. To better meet Halal requirements for proper and complete bleeding of slaughtered animals in order to obtain “spiritual quality meat”, the slaughter position on the neck recommended by Gregory et al. [27] should be considered for adoption in Halal industrial meat production. Indeed, the suggested position is not in contravention of the Halal religious requirements, given that the exact position of the neck cut has not been specified.

It is surprising that the percentage of rejected carcasses owing to the presence of both reflexes (independent and consequential) was higher for Operator C1 (14%) than for Operators C4 and C2 (10.25% and 9.75%, respectively). This confirms the need to decrease the reflexes consequential to false aneurysms, given that the other percentage cannot be monitored because we do not know the cause. Moreover, the same study highlights that the three operators showed a very similar percentage of rejected carcasses owing to the presence of reflexes consequential to false aneurysms (Table 5). As a consequence of the presence of such reflexes, the operators were forced to proceed with an irreversible electrical stunning, which led to the exclusion of the carcass from the Halal market and its sale as an animal slaughtered according to the traditional method. Animals with false aneurysms are still classified and accepted as Halal after corrective action (second cut of the neck), while cattle presenting rhythmic breathing and/or eye response are not accepted as Halal in this slaughterhouse. Indeed, the killing of an animal through stunning, whether mechanical or electric, is not contemplated by the requirements of the Halal certification bodies of the slaughterhouse. Therefore, we are faced with an ethical problem because carcasses judged unsuitable for consumption by the Islamic community for religious reasons are sold as meat from animals slaughtered in the traditional way [32]. This issue highlights the presence of a legislative gap regarding the production of meat destined for Islamic religious communities because its free consumption occurs without any kind of indication regarding slaughter. This situation is currently very common and emphasizes, once again, the lack of information about the method by which the animals are slaughtered and, more generally, the lack of knowledge about the food chain [32].

Gregory et al. [13] attributed a key role in false aneurysm onset not only to the point along the neck at which the cut is performed, but also to how the cut is made (force of the cut) and to the sharpness of the knife used. It implies that it is crucial to use a proper knife during Halal slaughter as animal welfare is also affected by the size of the knife. Moreover, the same study [13] showed that false aneurysms did not occur following electrical stunning with cardiac arrest. This indicates that aneurysms do not form when blood pressure is suppressed, but are present only during the early part of the bleeding period in animals whose heart is still beating. Taking into account these previous findings, it is clear that reversible stunning methods should be introduced during religious slaughtering in order to reduce the pain and distress caused by the act of slaughter. In fact, any stunning methods that do not cause the death of the animal, but render it only unconscious, are in line with Islamic principles [33].

## 5. Conclusions

The findings of the study show that performing the cut at the C1 and C2 sections of the neck reduced false aneurysm formation in comparison with C4. This is important evidence given that false aneurysms could extend the consciousness of Halal-slaughtered animals without pre-stunning, resulting in unnecessary suffering and welfare abuse [34] and prolonging the animal's state of consciousness owing to a failure to properly bleed. One way to control this risk would be to carry out pre-cut or post-cut stunning when the neck cut is performed at C4 level. While demonstrating that the cutting of the arteries at the level of the C1 or C2 vertebrae reduces the frequency of arrest of blood flow and, therefore, reduces the period of animal’s consciousness, this measure relative to the point of the cut does not resolve all potential causes of pain and distress that occur during ritual slaughter. In fact, it would also be important to reduce the period of animal’s consciousness independent of false aneurysms and related to the intrinsic characteristics of the animal. This period could certainly be decreased by introducing, during religious slaughter, a reversible stunning method that temporarily renders the animal unconscious, in agreement with Islamic principles.

## Figures and Tables

**Table 1:** Percentage of false aneurysm (FA) presence and their resolution.

Observations	Operator C4	Operator C2	Operator C1
Total number of animals	400	400	400
Total number of FA	41	29	29
Percentage of FA out of the total number of animals	10.25%	7.25%	7.25%
Number of resolved FA	23	12	13
Percentage of resolved FA out of total	56.10%	41.38%	44.83%
Number of unsolved FA	18	17	16
Percentage of unsolved FA out of total	43.90%	58.62%	55.17%

**Table 2:** Analysis of variance of the total number of false aneurysms (FA).

Groups	Source of Variation	Sum of Squares	Df	Fisher’s F	*p*-Value
Operators C4 and C2	Between groups	661.25	1	98.12	<0.001
	Within the groups	121.3	18		
	Total	782.55	19		
Operators C4 and C1	Between groups	720	1	89.50	<0.001
	Within the groups	144.8	18		
	Total	864.8	19		
Operators C2 and C1	Between groups	1.25	1	0.185	0.672
	Within the groups	121.3	18		
	Total	122.55	19		

**Table 3:** Analysis of variance of the resolved FA.

Groups	Source of Variation	Sum of Squares	Df	Fisher’s F	*p-*Value
	Between groups	605	1	75.21	<0.001
Operators C4 and C2	Within the groups	144.8	18		
	Total	749.8	19		
	Between groups	500	1	62.15	<0.001
Operators C4 and C1	Within the groups	144.8	18		
	Total	644.8	19		
	Between groups	5	1	0.622	0.441
Operators C2 and C1	Within the groups	144.8	18		
	Total	149.8	19		

**Table 4:** Analysis of variance of the unsolved FA.

Groups	Source of Variation	Sum of Squares	Df	Fisher’s F	*p-* Value
	Between groups	0.2	1	0.023	0.878
Operators C4 and C2	Within the groups	150.8	18		
	Total	151.0	19		
	Between groups	20	1	2.486	0.132
Operators C4 and C1	Within the groups	144.8	18		
	Total	164.8	19		
	Between groups	16.2	1	1.933	0.181
Operators C2 and C1	Within the groups	150.8	18		
	Total	167.0	19		

**Table 5:** Presence of rhythmic breathing (RB) and eye response (ER) or both (RB + ER) independent of and/or consequential to the formation of a false aneurysm (FA).

Observations	Operator C4	Operator C2	Operator C1
Total number of RB, ER, or RB + ER	41	39	56
Total RB, ER, or RB + ER independent of FA	23	22	40
Total RB, ER, or RB + ER consequential to FA	18	17	16
Operator error in performing the second cut of the blood vessel in order to resolve FA	4.5%	4.25%	4%
Rejection rate of carcasses independent and consequential to FA	10.25%	9.75%	14%
